# Factors Influencing Disabled Stroke Survivors’ Quality of Life in Rural China: Based on the Structural Characteristics and Psychometric Properties of the SF-36 Assessment

**DOI:** 10.3390/jcm12083012

**Published:** 2023-04-20

**Authors:** Qi Xu, Dingzhao Zheng, Shanjia Chen, Yiqi He, Zhenguo Lin, Dong Yao, Jiamei Wang, Jiapei Zhao, Longqiang Wu, Qiuju Liao, Yun Zhang, Tiebin Yan

**Affiliations:** 1 Department of Rehabilitation Medicine, Fifth Hospital of Xiamen, Xiamen 361101, China; 2 Department of Clinical Medicine, Xiamen Medical College, Xiamen 361101, China; 3 Department of Rehabilitation Medicine, Sun Yat-sen Memorial Hospital, Sun Yat-sen University, Guangzhou 510120, China; 4 The Engineering Technology Research Center of Rehabilitation and Elderly Care of Guangdong Province, Guangzhou 510120, China

**Keywords:** stroke survivors, physical disability, quality of life, dimension structure, psychometric tests, short form 36 assessment, rehabilitation exercise

## Abstract

Many stroke survivors’ quality of life is impaired. Few studies of factors influencing their quality of life have been based on the factors tested by the short form 36 instrument. This study did so with 308 physically disabled stroke survivors in rural China. Principal components analysis was applied to refine the dimension structure of the short form 36 assessment, followed by backward multiple linear regression analysis to determine the independent factors influencing quality of life. The structure revealed differed from the generic structure in showing that the mental health and vitality dimensions are not unidimensional. Subjects who reported access to the outdoors as convenient demonstrated better quality of life in all dimensions. Those who exercised regularly achieved better social functioning and negative mental health scores. Other factors influencing a better quality of life in terms of physical functioning were younger age and not being married. Being older and better educated predicted better role-emotion scores. Being female correlated with better social functioning scores, while men scored better on bodily pain. Being less educated predicted higher negative mental health, while being less disabled predicted better physical and social functioning. The results suggest that the SF-36’s dimension structure should be re-evaluated before using it to assess stroke survivors.

## 1. Introduction

Stroke is the leading cause of disability in China [1] and globally [2,3]. Due to population aging and improved stroke survival rates, the number of stroke survivors with disabilities is increasing worldwide [3,4]. Consequently, the burden of stroke is rising globally, especially in low-income countries [2]. China has the highest burden of stroke in the world, especially in its rural areas [5]. Most stroke survivors experience long-term dysfunction; the most common dysfunction is a physical disability with an accompanying decline in their quality of life [6]. It is, therefore, important to understand what that quality of life is and what factors may affect it. Clarifying this will help society develop better primary prevention and interventions.

When stroke survivors return home to live in the community, they often face difficulties adapting their impaired functioning to outdoor challenges, such as uneven terrain [7]. In this study, “outdoor convenience” refers to feeling comfortable and not too challenged functioning outdoors and getting where one wants to go. Recently, a study led by Twardzik found that those living in a friendly environment had a better quality of life in physical terms [8]. Another study by Stretton showed that stroke survivors had significant psychological benefits (a mental aspect of quality of life) from walking outdoors after carefully planning routes, building confidence, and developing routines [9]. However, there has been little, if any, research on how outdoor convenience affects quality of life after a stroke, especially in rural China. Therefore, this study was designed to explore to what extent outdoor convenience influences multiple dimensions of life quality measured using the short form 36-item health survey (SF-36).

Physical exercise could also influence stroke survivors’ quality of life. A study by Kwon et al. that investigated 575 stroke survivors using Korea’s National Health and Nutrition Examination Survey found that those who lacked regular physical exercise had a poorer quality of life [10]. Ali’s group meta-analyzed the effect of exercise on quality of life in 30 randomized and controlled studies of stroke survivors and found that exercise was related to moderate improvements in physical and mental health but had little impact on the social or cognitive aspects of life quality [11]. They also found that the beneficial effects of exercise for quality of life seemed to diminish during longer-term follow-up (3 to 9 months later) in a few studies, so they suggest that beneficial behavior change, opportunity, motivation to participate, and capability should all be considered in aiming for longer-lasting effects of exercise [11]. However, how those factors might go hand-in-hand with exercise to affect quality of life has been little studied. This study, therefore, examined multiple social factors and clinical characteristics beyond exercise that may interact to influence quality of life: outdoor convenience, duration since stroke onset, disability severity, and demographic characteristics. The term “rehabilitation exercise” in this study refers to physical exercise aiming to rehabilitate physical functioning.

### The SF-36 and Stroke Survivors

The SF-36 is a very popular tool for assessing health-related quality of life among stroke survivors [12]. It contains 36 items, 35 of which form 8 sub-scales or dimensions: physical functioning, role limitation in physical activity (role-physical), role limitation in emotional interactions (role-emotion), social functioning, bodily pain, mental health, vitality, and general health [13], as shown in Table A1 of Appendix A. The 36th item is health status compared with the previous year [13]. The SF-36 has been confirmed as having very good psychometric properties, with high internal consistency and good test-retest reliability with persons of different ages in various states of health [14,15,16].

However, some scholars believe that the factor structure should be explored in different populations to avoid abuse of the SF-36 [17,18]. In addition, studies have shown that the psychometric properties of SF-36 data from stroke survivors differ from those from other populations [19,20]. Therefore, the appropriateness of the dimensions needs further testing with stroke survivors.

This has been the first published study to determine a factor structure for the SF-36 by psychometric evaluation of stroke survivors in China. It also might be the first to explore comprehensively the factors that could predict the evaluation of each SF-36 dimension with stroke survivors.

## 2. Materials and Methods

### 2.1. Participants

This study involved a cross-sectional survey conducted in the Xiang’an villages and towns (the rural area) of Xiamen city in southern China. It was administered between May and December 2019. The subjects were community-dwelling stroke survivors with physical disabilities but normal intellectual ability (an Abbreviated Mental Test score ≥6 [21]).

The survey was administered by medical workers, liaison officers for the disabled and volunteers, all of whom received preparatory training beforehand. There were two steps to the survey. General information was collected in the subjects’ homes and the offices of the Disabled Persons Federation in the first step. The SF-36 was administered, and other relevant rehabilitation information was collected face-to-face in the second step, with guidance from the medical staff. If a subject was illiterate or unable to write, the responses were filled in by the medical staff with explanations of the items.

**Figure 1:**
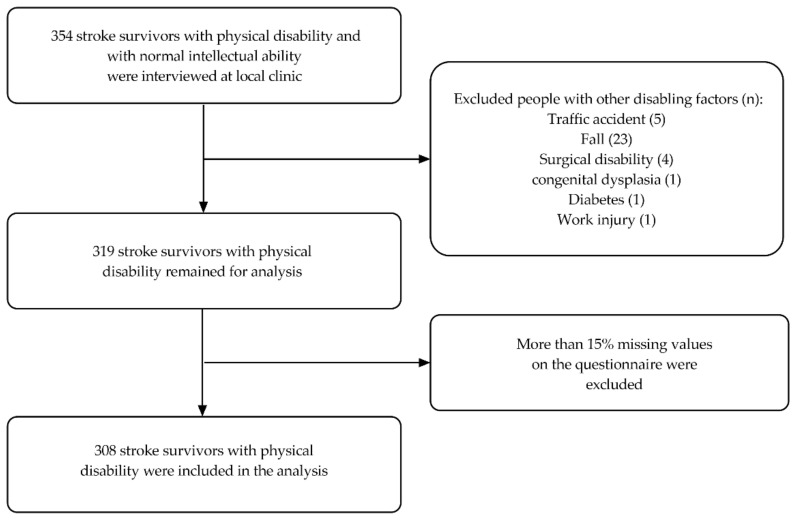
Inclusion and Exclusion Flowchart.

All stroke survivors with physical disabilities and normal intellectual ability in that district were initially considered for investigation. 354 stroke survivors were interviewed at local clinics. Those with a factor other than stroke contributing to their disability and those with more than 15% missing values on the questionnaire were excluded. Finally, 308 subjects were included in the analysis (see Figure 1). The demographics of the 308 stroke survivors studied are described in Table 1.

### 2.2. Assessment Instrument Scaling

The study applied the Chinese version of the SF-36 based on American norms [22,23]. The assessment’s scaling was reset so that a higher score consistently indicated better functioning or status. Outdoor convenience was quantified by asking, “Can you easily get where you want to go?” with very inconvenient (1), inconvenient (2) and convenient (3) as the response choices. Disability severity was evaluated based on China’s national standards for the classification and severity grading of people with disability. Its grades are extremely severe (totally dependent) (1), severe (basically dependent) (2), moderate (partially dependent) (3) and mild (independent) [24]. Routine rehabilitation exercise was quantified by asking each subject whether they routinely perform physical exercise for rehabilitation purposes. The replies were never (scored as 1), sometimes (2) or usually (3). Age and duration since stroke elicited numeric responses. Other replies were quantified as shown in Table 1 (e.g., Male as (1) and Female as (2). Employment status was not quantified since the employment rate (2.6%) was too small to analyze.

### 2.3. Statistical Analyses

All data were analyzed with version 22 of the SPSS software suite. The factor structure was determined by exploratory factor analysis using principal component analysis to extract the factors and varimax rotation. The Kaiser–Meyer–Olkin measure and Bartlett’s test of sphericity tested the adequacy of the data for factor analysis. According to Kaiser’s criterion, eigenvalues greater than 1 indicate useful factors or dimensions. Factor loadings equal to or more than 0.4 were considered significant [25].

To obtain standardized dimension scores, the sum of each dimension was linearly transformed to a 0–100 scale, with a higher score indicating better quality of life. The transformed dimension score = 100 × [(actual raw score − lowest possible raw score)/possible raw score range] [26,27]. A Cronbach’s alpha coefficient of 0.6 or greater indicated good internal reliability of the scale [28]. The distributions of all of the dimension scores were checked for floor and ceiling effects. Such an effect was deemed present if more than 70% of the respondents obtained the lowest or highest possible score [29].

Multiple linear regressions (backward regression) were evaluated to assess independent risk factors influencing quality of life. The SF-36 dimensions were considered the dependent variables. The predictors included in the regression models were outdoor convenience, routine rehabilitation exercise, disability severity, duration since stroke, age, gender, marital status, and education. *p* ≤ 0.05 was accepted as indicating statistical significance, and standard beta coefficients were recorded.

## 3. Results

### 3.1. The Validity of the SF-36’s Factor Structure

**Figure 2:**
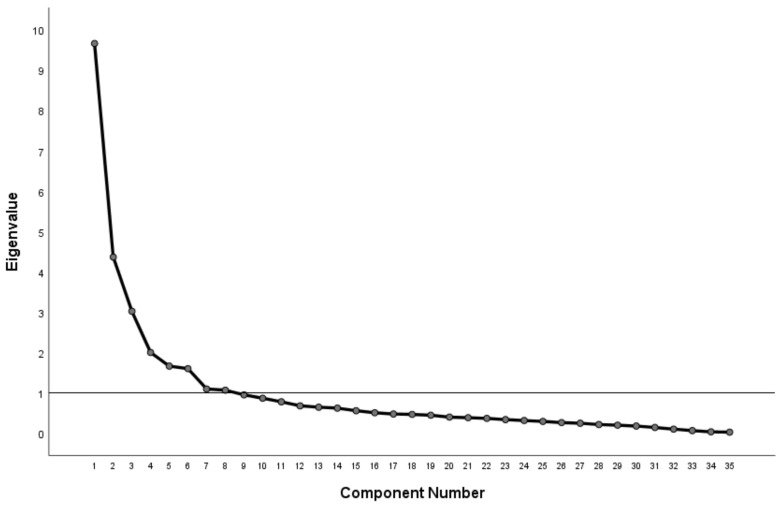
The components’ eigenvalues.

The SF-36’s factor structure was tested using the data collected. The Kaiser–Meyer–Olkin statistic was 0.848 (so >0.7), and Bartlett’s test of sphericity (*p* ≤ 0.001) indicated that the data set was adequate and appropriate for factor analysis. The principal component factor analysis yielded eight factors (dimensions) with an eigenvalue greater than 1. The plot of components associated with the eigenvalues is shown in Figure 2. Those factors accounted for 69.9% of the variance, with the item loadings ranging from 0.573 to 0.966, indicating that all the items in the model were valuable. The valid SF-36 factor structure (Chinese version) is shown in Table 2. This is not exactly the same as the standard eight factors (shown in Table A1 of Appendix A), which contain Vitality and Mental health dimensions for a normal population [13].

### 3.2. Reliability of the SF-36’s Scales

The internal consistency of each dimension was examined by computing Cronbach’s alpha coefficients. They ranged from 0.648 (Bodily pain) to 0.985 (Role-emotion) (all >0.6), indicating good internal consistency for the specific items. Three dimensions had floor effects, with more than 70% of the respondents obtaining the lowest possible score: physical functioning, role-physical, and role-emotion [29]. This is consistent with the findings of previous research [18,20]. The internal consistency and the floor and ceiling effects are presented in Table 3.

### 3.3. Multivariate Analysis for Independent Risk Factors Influencing Quality of Life

The impact of the factors influencing each dimension of quality of life was assessed using backward regression analysis (Table 4). The results show that outdoor convenience positively influenced all life quality dimensions (β_min_ = 0.124, β_max_ = 0.347, *p* ≤ 0.05). Routine rehabilitation exercises positively influenced social functioning (β = 0.169, *p* ≤ 0.01) and negative mental health (β = 0.186, ≤0.01) but was not significantly related to the other dimensions. Less severe disability, younger age and being unmarried were significantly associated with better physical functioning, while older age and more education were positively related to role-emotion. Being a less disabled woman predicts better social functioning. Being male predicts less bodily pain. Being less educated correlates with a better negative mental health score (a higher score indicates better).

## 4. Discussion

### 4.1. The New Factor Structure

Although since the 1980s, the SF-36 has been widely used with eight factors to measure health-related quality of life [30], critics have been calling for factor structure analysis for stroke survivors [17,18,19,31]. Dallmeijer’s group conducted an exploratory factor analysis and reported that with a stroke population, the vitality dimension lacks unidimensionality since the items are split up and loaded on positive and negative aspects of health status [19]. This study was the first to apply factor analysis of the SF-36 among rural Chinese stroke survivors, and it has confirmed that. It also found that mental health splits into positive and negative aspects. These findings highlight the importance of factor structure evaluation and emphasize that taking the SF-36’s generic factor structure as generally applicable is inappropriate. If items in each dimension do not cluster as a common underlying construct, it is not legitimate to combine them to generate dimension scores [32]. This study has been the first to explore the factors influencing stroke survivors using the new SF-36 dimension structure.

At the beginning of the 20th century, scholars proposed that clinical mental health has two sides (positive and negative characteristics) that can present separately, and their roles could differ [33,34]. Positive mental health is associated with subjective well-being and affective balance, which maximizes the realization of one’s potential and promotes participation. In contrast, negative mental health is associated with reduced functional capacity and/or symptoms which may cause considerable health burden [35]. The two aspects of mental health should therefore be recognized and treated separately in the clinic; the results of the present study support this reasoning. In addition, the dual nature of self-reported mental health should be distinguished in stroke survivors with physical disabilities.

In addition, the study’s data shows that stroke survivors with physical disabilities had relatively low scores on all quality of life dimensions, with a median range of 0–52 (100 representing normal functioning). Physical functioning, role-physical and role-emotion, in particular, showed significant floor effects, consistent with previous studies with a stroke survivor population [18,20]. A floor effect reflects the value beyond which the dimension has limited sensitivity for detecting worsening functioning, and clinical improvement may not be reflected in score changes [18]. The SF-36 was developed aimed toward less disabling medical conditions. However, stroke patients often have substantial impairment in these dimensions compared with the normal population. It is, therefore, necessary to develop these three dimension scales to make them more appropriate for a stroke population functioning poorly in these domains based on examining the scales’ items and item response options [18].

### 4.2. Clinical Application of the New SF-36 Factor Structure

Among the three dimensions showing floor effects, physical functioning is closely related to the existing idea of disability severity and probably not easy to improve. Role-physical and role-emotion reflect one’s belief in their ability to accomplish something. They are aspects of self-perceived behavior control [36], so they might be improved by intention or behavior change according to the theory of planned behavior [37]. Other dimensions may similarly find improvement possibilities by considering behavior or other alterable factors. Li’s group has shown that intervention integrating health belief and planned behavior theory can improve the quality of life of stroke patients [38]. There has, however, been little research investigating specific behaviors in each dimension impacting stroke survivors’ quality of life.

This study explored outdoor convenience and routine rehabilitation exercises as two potentially-important factors. The data indicates that outdoor convenience significantly affects each dimension of quality of life, so it would be a good place to start. In recent years, a few trial initiatives, like the We Walk program, have sought to reduce sedentary behavior and increase physical activity among stroke survivors [39,40]. Such initiatives could be relevant to outdoor convenience. Outdoor programs of that sort should build confidence and thus feelings of outdoor convenience. 

Much evidence shows that physical exercise protects against negative mental health [41,42]. However, limited studies have reported physical exercise’s impact on positive mental health [43]. White and his colleagues report that exercise in transport or at leisure improves positive mental health [44], but routine rehabilitation exercise was not helpful in this study. That may be because the majority of the respondents seldom enjoy leisure-related or travel-related exercise. Only 12% reported feeling that getting outdoors was convenient. It may also be because stroke survivors more readily identify the negative aspects of their situation than the positive aspects. The results nevertheless suggest that outdoor exercise should be promoted because of its effects on negative mental health. Unexpectedly, routine rehabilitation exercises did not independently improve physical functioning. That could be because exercise effects diminish over time, as a previous longer-term study has suggested [11].

Some factors are, of course, unalterable. The data from this study do not show that increasing duration since the stroke predicts significant improvement in either dimension of quality of life. It is, therefore, important to help stroke survivors without waiting for autogenous improvement. The data also show that the severity of disability affects physical and social functioning but not other dimensions. That is consistent with the paradox of disability: two individuals with the same severity of structural and functional impairment may demonstrate different levels of disability since disability is determined by both behavior and pathology [45]. Stroke survivors should be encouraged to improve their overall quality of life as much as possible, whatever their level of disability.

That physical functioning degrades with age is well understood [12]. But role-emotion is not necessarily degraded, as older stroke survivors might cope better psychosocially since they have less expectation of good health [46]. Women demonstrated better social functioning in this study, which may be a China-specific finding. In China, group dancing is very popular, and the majority of the participants are women. This sort of group activity (tai chi is another example) may promote women’s recovery. Being married predicts poorer physical functioning. This is consistent with the findings of previous research: spouses may be overprotective and underestimate the utility of physical activity [47,48]. Males reported less bodily pain. This may be explained by women having greater pain sensitivity than men [49]. More education predicts better role-emotion results, probably due to education fostering flexible coping skills and making the survivors more adaptable to challenges [50]. More education is negatively associated with negative mental health (a higher score indicates better), which might be because such people are more sensitive and detect and report negative feelings more readily.

### 4.3. Limitations of the Study

It is, of course, important to recognize this study’s very special population. First, it was limited to stroke survivors with a physical disability living in rural southern China. The general relevance of the findings remains to be demonstrated. Secondly, it is important to bear in mind that this was a cross-sectional study. Any causal relationships remain to be demonstrated. Thirdly, all of the data were self-reported. Although the investigators who administered the survey had received preparatory training, recall bias, socially-acceptable responding and exaggeration were all probably operative to some extent. In addition, note too that the three dimensions with floor effects have limited value, which could underestimate the factors influencing quality of life.

## 5. Conclusions

The SF-36’s dimension structure should be re-evaluated before exploring factors influencing its ratings of stroke survivors. A new factor structure for the SF-36 assessment was defined as applicable in particular to stroke survivors living in rural China. Negative and positive mental health must be distinguished in the dimension structure and the clinical application of the assessment. Three dimensions showed floor effects, indicating a need to develop more appropriate and useful scales for stroke survivors assessing those dimensions.

Influencing factors were identified based on the new structure. Outdoor convenience affected each dimension of quality of life, and routine rehabilitation exercises affected social functioning and negative mental health. They were confirmed to play an essential role in influencing quality of life. Therefore, multidisciplinary cooperation among rehabilitation clinicians, social workers, recreation therapists, behavior therapists and so on is important. Quality of life does not necessarily improve with time after a stroke. The severity of disability affects only a few dimensions of quality of life, which suggests that the whole course and specific dimensions of a stroke survivor’s rehabilitation should be carefully executed. Furthermore, different demographic characteristics affect other dimensions of quality of life, emphasizing that rehabilitation should be individualized. Interventions to improve quality of life can be developed based on the factors’ different effects.

## Figures and Tables

**Table 1:** Demographic characteristics of the 308 participants.

Categorization	N (%)	Missing Records (N)
Sex		1
Male (1)	166 (54.07%)	
Female (2)	141 (45.93%)	
Age	68.70 ± 12.10	14
24–59 years	66 (21.78%)	
60–69 years	90 (29.70%)	
70–79 years	76 (25.08%)	
≥80 years	62 (20.46%)	
Education		2
None (1)	108 (35.29%)	
Elementary (2)	128 (41.83%)	
Secondary or more (3)	70 (22.88%)	
Marital status		2
Unmarried (1)	116 (37.91%)	
Married (2)	190 (62.09%)	
Employment		2
Employed	298 (97.39%)	
Unemployed	8 (2.61%)	
Routine rehabilitation exercise		5
Never (1)	144 (47.52%)	
Sometimes (2)	128 (42.24%)	
Usually (3)	31 (10.23%)	
Outdoor convenience		0
Very inconvenient (1)	155 (50.32%)	
Inconvenient (2)	117 (37.99%)	
Convenient (3)	36 (11.69%)	
Disability severity		9
Extremely severe (totally dependent) (1)	33 (11.04%)	
Severe (basically dependent) (2)	240 (80.27%)	
Moderate (partially dependent) (3)	10 (3.34%)	
Mild (independent) (3)	16 (5.35%)	
Duration since stroke	10.50 ± 6.77	5
0–4 years	73 (24.09%)	
5–9 years	108 (35.64%)	
10–14 years	70 (23.10%)	
15–19 years	26 (8.58%)	
≥20 years	26 (8.58%)	

**Table 2:** The resulting 8-factor structure for the SF-36 assessment for stroke survivors with physical disability.

	Factor 1	Factor 2	Factor 3	Factor 4	Factor 5	Factor 6	Factor 7	Factor 8
Ⅰ Physical functioning								
Vigorous activities	0.677	0.177	0.074	−0.008	−0.168	0.117	0.144	0.097
Moderate activities	0.708	0.005	0.420	0.055	0.152	0.106	−0.092	0.101
Lifting or carrying groceries	0.809	0.045	0.231	0.053	0.118	0.066	−0.065	0.072
Climbing several flights of stairs	0.784	0.006	0.133	0.029	0.000	0.080	0.145	0.028
Climbing one flight of stairs	0.832	0.033	0.257	0.098	0.143	0.091	−0.009	0.066
Bending, kneeling or stooping	0.814	0.070	0.002	0.043	0.005	0.044	0.096	0.101
Walking more than 1500m	0.823	0.039	0.000	−0.001	0.070	0.071	0.116	−0.038
Walking several blocks	0.817	0.030	0.112	0.035	0.097	0.168	−0.023	0.019
Walking one block	0.746	0.040	0.225	0.082	0.133	0.112	−0.094	0.195
Bathing or dressing yourself	0.681	0.104	0.290	−0.002	0.086	−0.069	−0.090	0.240
Ⅱ Role-physical (RP)								
RP-Cut down the amount of time in work or other activities	0.371	−0.006	0.763	0.074	0.199	0.031	−0.044	0.044
RP-Accomplished less than you would like	0.209	0.003	0.879	0.044	0.001	0.016	0.099	0.100
RP-Limited in the kind of work or other activities	0.214	0.002	0.901	0.125	−0.021	0.059	0.065	0.029
RP-Had difficulty performing the work or other activities	0.321	0.000	0.790	0.154	−0.026	−0.006	0.105	0.045
Ⅲ Role-emotion (RE)								
RE-Cut down the amount of time in work or other activities	0.031	0.056	0.110	0.963	0.086	0.044	0.041	0.026
RE-Accomplished less than you would like	0.103	0.054	0.125	0.952	0.143	0.035	0.074	0.039
RE-Didn’t do work or other activities as carefully as usual	0.077	0.050	0.107	0.966	0.116	0.045	0.051	0.024
Ⅳ Social functioning								
Extent that physical health or emotional problems interfered with normal social activities	0.304	0.075	0.113	0.137	0.036	0.203	−0.038	0.756
Time that physical health or emotional problems interfered with social activities	0.401	0.249	0.155	−0.058	0.159	0.060	0.157	0.657
Ⅴ Bodily pain								
Bodily pain	0.026	0.116	0.090	0.153	0.247	−0.007	0.750	−0.168
Pain interfering with normal work	0.096	−0.058	0.093	0.008	0.083	0.003	0.822	0.190
Ⅵ Positive mental health								
Feel full of pep	0.188	0.248	0.020	0.072	0.280	0.586	0.066	0.157
Calm and peaceful	0.114	0.331	0.038	0.036	−0.057	0.771	0.035	−0.020
Have a lot of energy	0.130	0.005	0.005	−0.012	0.240	0.697	0.071	−0.016
Happy	0.126	0.112	0.028	0.076	0.184	0.698	−0.149	0.204
Ⅶ Negative mental health								
Very nervous	0.037	0.627	−0.062	0.065	−0.060	0.173	0.046	−0.097
Down in the dumps	0.071	0.811	0.001	−0.013	0.098	0.133	−0.022	0.027
Downhearted and blue	0.114	0.787	0.071	0.078	0.191	0.119	−0.065	0.019
Feel worn out	0.066	0.783	0.002	0.084	0.173	0.031	0.075	0.120
Feel tired	0.035	0.718	−0.017	−0.055	0.229	0.058	0.046	0.260
Ⅷ General health								
Get sick a little easier than other people	0.090	0.236	−0.019	0.142	0.573	0.145	0.266	−0.016
Expect health to get worse	−0.016	0.170	−0.025	0.148	0.697	0.091	0.225	0.132
As healthy as anybody	0.079	0.344	0.175	−0.084	0.551	0.364	−0.053	−0.093
Excellent health	0.036	0.400	0.225	0.045	0.630	0.234	−0.045	0.000
Health status	0.194	−0.025	−0.018	0.113	0.616	0.091	0.027	0.079

Note: the bold numbers indicate items clustered in the specific dimension.

**Table 3:** Internal consistency, floor and ceiling effects of the SF-36.

Sub-Scales	Cronbach’s Alpha	Floor %	Ceiling %	Median	Minimum Score	Maximum Score
I Physical functioning (PF)	0.933	76.62%	0.32%	0	0	85
II Role-physical (RP)	0.912	94.16%	2.60%	0	0	100
III Role-emotion (RE)	0.985	70.45%	26.62%	0	0	100
IV Social functioning (SF)	0.669	30.52%	0.32%	11	0	100
V Bodily pain (BP)	0.648	2.92%	6.17%	52	12	100
VI Positive mental health (PMH)	0.749	3.25%	0.32%	25	0	100
VII Negative mental health (NMH)	0.838	0.32%	0.32%	42	0	100
VIII General health (GH)	0.758	5.84%	0.32%	20	0	100

Notes: N = 308. The Chinese version of the assessment.

**Table 4:** Backward regression results indicate factors influencing SF-36 evaluation.

QoL Dimension	Significant Predictors	Standard Beta Coefficient	*p*
I Physical functioning (PF)	Outdoor convenience	0.327	<0.001
	Age	−0.227	<0.001
	Disability severity	0.137	0.001
	Marital status	−0.137	0.015
II Role-physical (RP)			
	Outdoor convenience	0.219	<0.001
III Role-emotion (RE)			
	Outdoor convenience	0.259	<0.001
	Age	0.205	0.002
	Education	0.168	0.011
IV Social functioning (SF)			
	Outdoor convenience	0.347	<0.001
	Rehabilitation exercise	0.169	0.003
	Disability severity	0.165	0.003
	Gender	0.124	0.022
V Bodily pain (BP)			
	Outdoor convenience	0.124	0.044
	Gender	−0.121	0.038
VI Positive mental health (PMH)			
	Outdoor convenience	0.193	<0.001
VII Negative mental health (NMH)			
	Outdoor convenience	0.219	<0.001
	rehabilitation exercise	0.186	0.003
	Education	−0.137	0.021
VIII General health (GH)			
	Outdoor convenience	0.222	<0.001

## Data Availability

All data and analyses can be requested from the corresponding author (T.Y.).

## Appendix A

**Table A1:** Generic factor structure of the SF-36 assessment for a normal population.

I Physical functioning
Vigorous activities
Moderate activities
Lifting or carrying groceries
Climbing several flights of stairs
Climbing one flight of stairs
Bending, kneeling or stooping
Walking more than a mile
Walking several blocks
Walking one block
Bathing or dressing yourself
II Role physical (RP)
RP-Cut down the amount of time on work or other activities
RP-Accomplished less than you would like
RP-Limited in the kind of work or other activities
RP-Had difficulty performing the work or other activities
III Role emotion (RE)
RE-Cut down the amount of time on work or other activities
RE-Accomplished less than you would like
RE-Didn’t do work or other activities as carefully as usual
IV Social functioning
Extent that physical health or emotional problems interfered with normal social activities
Time that physical health or emotional problems interfered with social activities
V Bodily pain
Bodily pain
Pain interfering with normal work
VI Mental health
Very nervous
Down in the dumps
Downhearted and blue
Happy
Calm and peaceful
VII Vitality
Feel full of pep
Have a lot of energy
Feel worn out
Feel tired
VIII General health
Get sick a little easier than other people
Expect health to get worse
As healthy as anybody
Excellent health
Health status
